# Characteristics and predictors of chronic critical illness in the intensive care unit

**DOI:** 10.5935/0103-507X.20190088

**Published:** 2019

**Authors:** Fernanda Perito Aguiar, Glauco Adrieno Westphal, Michelli Marcela Dadam, Elisa Cristina Correia Mota, Felipe Pfutzenreuter, Paulo Henrique Condeixa França

**Affiliations:** 1 Programa de Pós-Graduação em Saúde e Meio Ambiente e Departamento de Medicina, Universidade da Região de Joinville - Joinville (SC), Brasil.; 2 Unidade de Terapia Intensiva, Hospital São José - Joinville (SC), Brasil.

**Keywords:** Critical illness, Respiration, artificial, Pressure ulcers, Muscle weakness, Intensive care units

## Abstract

**Objective:**

To characterize patients with chronic critical illness and identify predictors of development of chronic critical illness.

**Methods:**

Prospective data was collected for 1 year in the intensive care unit of a general hospital in Southern Brazil. Three logistic regression models were constructed to identify factors associated with chronic critical illness.

**Results:**

Among the 574 subjects admitted to the intensive care unit, 200 were submitted to mechanical ventilation. Of these patients, 85 (43.5%) developed chronic critical illness, composing 14.8% of all the patients admitted to the intensive care unit. The regression model that evaluated the association of chronic critical illness with conditions present prior to intensive care unit admission identified chronic renal failure in patients undergoing hemodialysis (OR 3.57; p = 0.04) and a neurological diagnosis at hospital admission (OR 2.25; p = 0.008) as independent factors. In the model that evaluated the association of chronic critical illness with situations that occurred during intensive care unit stay, muscle weakness (OR 2.86; p = 0.01) and pressure ulcers (OR 9.54; p < 0.001) had the strongest associations. In the global multivariate analysis (that assessed previous factors and situations that occurred in the intensive care unit), hospital admission due to neurological diseases (OR 2.61; p = 0.03) and the development of pressure ulcers (OR 9.08; p < 0.001) had the strongest associations.

**Conclusion:**

The incidence of chronic critical illness in this study was similar to that observed in other studies and had a strong association with the diagnosis of neurological diseases at hospital admission and chronic renal failure in patients undergoing hemodialysis, as well as complications developed during hospitalization, such as pressure ulcers and muscle weakness.

## INTRODUCTION

Advances in intensive care and new technologies have resulted in high survival rates among critically ill patients. However, some patients who survive acute illnesses remain dependent on intensive treatment for long periods, and these patients constitute the chronic critical illness (CCI) population.^(1-3)^ This population is usually composed of patients of an advanced age and/or patients with chronic comorbidities who develop prolonged and continuous dependence on mechanical ventilation (MV) and complications such as cerebral dysfunction, muscle weakness, endocrine disorders, malnutrition and pressure ulcers.^(4)^

It is difficult to confirm the evolution from the acute phase to the chronic phase of a CCI because there is no obvious reference that defines this transition.^(3,5)^ In addition, there is no uniformly accepted definition to characterize CCI.^(5,6)^ The most commonly used definitions are the duration of the MV (> 14 or > 21 days) or the need for a tracheostomy.^(5,7)^ For parameterization purposes, a consensus previously defined CCI as a condition in which MV is prolonged and lasts for at least 21 days.^(8-10)^ Depending on the criteria used, patients with CCI constitute up to 15% of critical patients,^(9,11)^ and considering the increase in life expectancy and the prevalence of CCI, this population is expected to increase over the years.^(12-14)^

Patients with CCI constitute a population with specific pathophysiological characteristics, and up to 20% of the population suffer from neuroendocrine, metabolic and neuromuscular alterations.^(15,16)^ The clinical complications resulting from these changes imply a greater need for home care or institutionalization and lead to frequent rehospitalizations, high rates of hospital and post-hospital mortality, and high direct and indirect costs.^(7,17-22)^ Mortality after six months from hospital discharge exceeds the mortality of patients with the majority of malignancies, and the patients surviving with CCI infrequently return to social activities.^(23)^

Despite its relevance, information on the epidemiology of CCI is still scarce.^(16,24,25)^ In addition to a better pathophysiological understanding of critical illness chronification, the epidemiological characterization of patients with CCI can aid in the planning and implementation of care protocols that prevent the development of devastating conditions in terms of morbidity and mortality. This information can also support health teams in adequately communicating with family members about the short- and long-term perspectives and in providing decision support on the continuation of advanced life support therapies.^(3)^

Given this context, the purpose of this study was to characterize the CCI population to identify predictors of development of the CCI state by comparing patients with CCI to other mechanically ventilated patients.

## METHODS

This observational study was performed from August 2015 to July 2016 in a general intensive care unit (ICU) with 14 beds in a South-Brazilian public hospital without long-term care facilities. The hospital is a regional referral center for emergency, urgency, neurology, neurosurgery, oncology, orthopedic, traumatology, burn and transplant cases.

The research project was evaluated and approved by the Research Ethics Committee of *Universidade da Região de Joinville* (UNIVILLE), under opinion 1.153.723. Informed consent was voluntarily provided by all subjects included in the study (or their legal guardians).

All subjects older than 18 years who needed MV for more than 48 hours were included. Patients for whom a spinal cord injury or a neuromuscular disease (Guillain-Barré syndrome, myasthenia gravis, amyotrophic lateral sclerosis and multiple sclerosis) was the primary cause of hospitalization, patients who required emergency tracheostomy and patients who were diagnosed with brain death were not included; in addition, patients readmitted to the ICU, those with incomplete medical records and those who were transferred to another hospital were excluded. The subjects included were followed daily from the time of admission to the ICU to the time of hospital discharge or hospital death.

The patients were divided into two groups: the CCI Group, which included subjects who required MV for at least 21 days^(7,8,16)^ and/or tracheostomy to facilitate weaning of the MV,^(6)^ and the Control Group, which included patients who required MV for fewer than 21 days.

The data were collected prospectively from the subjects' electronic medical records and transferred to a data collection form. The following information were recorded: demographic information (age, sex, skin color, date of hospital admission, date of ICU admission, date of discharge from the ICU and hospital discharge date or date of death); previous comorbidities (diabetes mellitus, systemic arterial hypertension, congestive heart failure, chronic lung disease, chronic renal failure - CRF - being treated by hemodialysis, malignant neoplasm, ischemic heart disease and human immunodeficiency virus (HIV) disease); diagnosis related group at hospital admission and at ICU admission, including those related to the following neurologic conditions: ischemic and hemorrhagic stroke, subarachnoid hemorrhage, neoplasm affecting the central nervous system, and traumatic brain injury; procedures and medicines administered during the first 21 days in the ICU (MV start date, number of successful extubations - more than 48 hours with spontaneous breathing, date of tracheostomy, medications used during hospitalization - corticosteroids, neuromuscular relaxant, aminoglycosides - and unscheduled surgery during ICU stay; and comorbidities during ICU stay (muscle weakness - grade 1 to 5 on the Medical Research Council - MRC scale, acute respiratory distress syndrome - ARDS; *delirium* according to the Confusion Assessment Method for the *Intensive Care Unit* - CAM-ICU*,* pressure ulcers grade 2 to 4, upper gastrointestinal bleeding, candidemia, ventilator-associated pneumonia and sepsis - presumed or confirmed infection plus at least one organ dysfunction).

To evaluate the muscular strength of the patients, we used the Medical Research Council (MRC) scale, which classifies muscle strength by five grades: grade 0 (absence of movement), grade 1 (only a trace or flicker of a contraction), grade 2 (active movement only when gravity is removed), grade 3 (active movement against gravity), grade 4 (active movement against resistance), and grade 5 (normal strength).^(26)^

The Simplified Acute Physiology Score 3 (SAPS 3)^(27)^ and the Charlson index^(24,28)^ were calculated on the first day of ICU admission. The Sequential Organ Failure Assessment (SOFA)^(29)^ was calculated on the first, seventh, fourteenth and twenty-first days of MV.

### Statistical analysis

The data were analyzed using MedCalc statistical software, version 16.4.3 (MedCalc Software BVBA, Ostend, Belgium). Continuous variables were expressed as means ± standard deviations. The Kolmogorov-Smirnov test was applied to assess the normal distribution of the data. We used Student's *t* test to compare means and the nonparametric Mann-Whitney U test to compare variables with asymmetrical distributions. Categorical variables were expressed as absolute and relative values and were compared using the chi-squared test. A p value < 0.05 was considered statistically significant. The odds ratio (OR) and 95% confidence interval (95%CI) were determined from the comparison of the two groups of subjects, which was performed to evaluate the impact of each variable on the development of CCI. All variables for which a significance level of p < 0.10 was obtained in the univariate model were selected to construct the three following models of multivariate analyses by logistic regression, considering CCI as a dependent variable: a global model, which assessed preexisting characteristics and those at admission to the ICU, as well as the characteristics developed during hospitalization; a model that analyzed only the preexisting characteristics and those at admission; and a model that exclusively analyzed the characteristics developed during hospitalization.

## RESULTS

**Figure 1:**
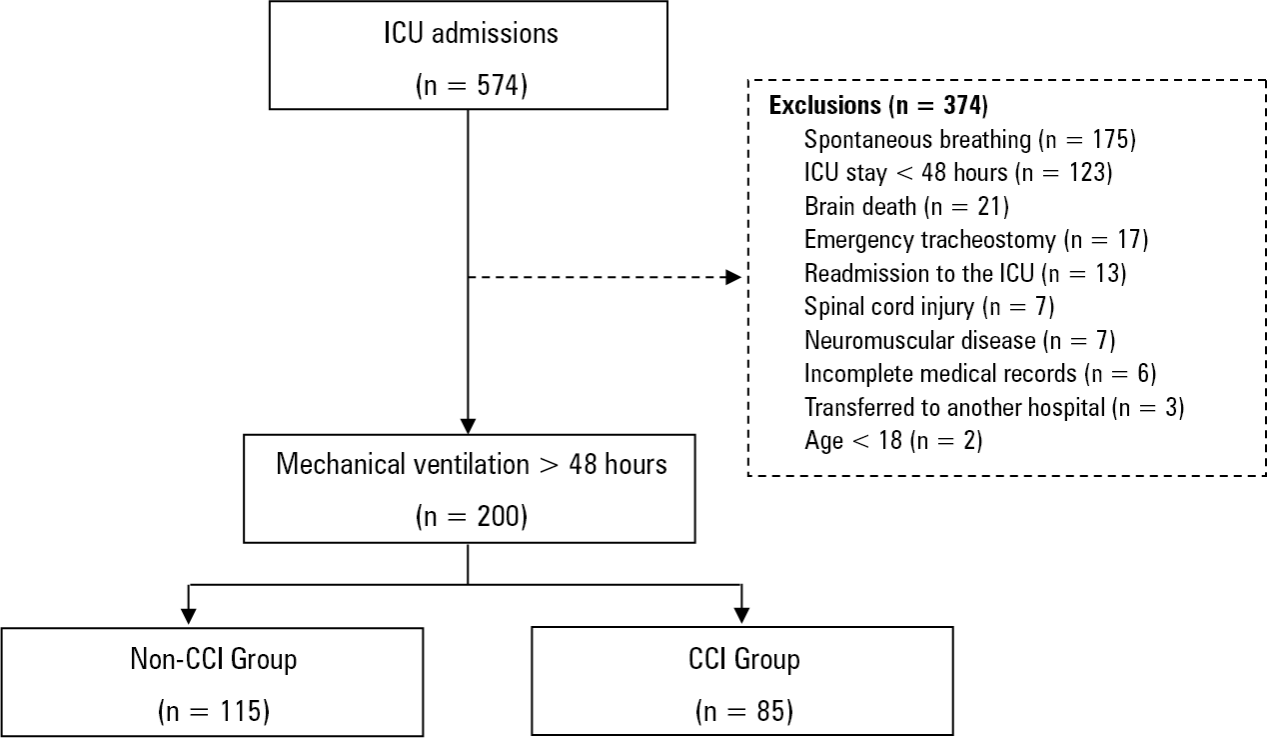
Study flowchart. ICU - intensive care unit; CCI - chronic critical illness.

During the study period, there were 574 hospitalizations in the ICU, and 374 did not meet the inclusion criteria. Among the 200 subjects analyzed, 85 were included in the CCI Group because they required ventilatory support for at least 21 days (n = 56) and/or tracheostomy for ventilatory support weaning (n = 29). The other 115 subjects were included in the Control Group (Figure 1). The subjects who were discharged from the ICU and readmitted afterwards (n = 3) were not reincluded in the analysis.

Table 1 compares the CCI Group to the Control Group in relation to the characteristics presented at hospital admission. Compared with the Control Group, the CCI Group had a higher mean age (p = 0.04), higher frequency of CRF (p = 0.003), higher frequency of neurological diseases as the diagnosis for hospital admission (p = 0.009), and a longer hospitalization time until ICU admission (p = 0.02). No differences were identified in relation to sex, ethnicity, the Charlson index, previous ICU hospitalization or surgical diseases.

**Table 1:** Characteristics of patients before intensive care unit admission

Characteristics	CCI Group (n = 85)	Control Group (n = 115)	p value
Age (years)	54.4 ± 17.5	49.3 ± 17.4	0.04
Male sex	56 (65.9)	72 (62.6)	0.63
Skin color			
White	75 (88.2)	98 (85.2)	0.54
Black	5 (5.9)	5 (4.35)	0.62
Brown	5 (5.9)	12 (10.4)	0.25
Previous comorbidities			
Diabetes mellitus	16 (18.8)	19 (16.5)	0.67
Systemic arterial hypertension	32 (37.6)	40 (34.8)	0.67
Congestive heart disease	9 (10.6)	6 (5.2)	0.15
Chronic lung disease	19 (22.4)	15 (13.0)	0.08
Chronic renal failure	13 (15.3)	4 (3.4)	0.003
Malignant neoplasm	5 (5.9)	9 (7.8)	0.59
Ischemic heart disease	4 (4.7)	3 (2.6)	0.42
AIDS	2 (2.4)	3 (2.6)	0.90
Charlson index	1.5 ± 1.3	1.4 ± 1.1	0.42
Diagnostic group at hospital admission			
Cardiovascular	3 (3.5)	6 (5.2)	0.57
Neurological	37 (43.5)	30 (26.1)	0.009
Respiratory	6 (7.1)	9 (7.8)	0.84
Gastrointestinal	4 (4.7)	12 (10.4)	0.14
Trauma	30 (35.3)	44 (38.3)	0.67
Acute renal dysfunction	1(1.1)	4 (3.5)	0.30
Malignant neoplasm	1(1.1)	4 (3.5)	0.30
Other diseases	5 (5.9)	6 (5.2)	0.83
Sector prior to ICU admission			
Other ICU	1 (1.1)	1 (0.9)	0.83
Inpatient units	45 (53.0)	68 (59.1)	0.38
Emergency room	39 (45.9)	46 (40.0)	0.41
Surgical and nonsurgical patient			
Surgical	57 (67.0)	75 (65.2)	0.79
Nonsurgical	28 (33.0)	40 (34.8)	0.79
Hospital length of stay prior to ICU admission (days)	8.1 ± 10.1	5.1 ± 7.0	0.02

CCI - chronically critically illness; AIDS - Acquired Immunodeficiency Syndrome; ICU - intensive care unit. Results expressed as n (%) or mean ± standard deviation.

Table 2 shows the comparison between the CCI Group and the Control Group in relation to the variables recorded at admission and during their stay in the ICU. There was no difference between the groups regarding the SAPS 3, the diagnoses for admission to the unit or the SOFA score at 7-day intervals. More individuals in the CCI Group than in the Control Group developed the following comorbidities during their ICU stay: muscle weakness (60.0% *versus* 14.8%; p < 0.001), pressure ulcers (68.2% *versus* 11.3%; p < 0.001), sepsis (44.7% *versus* 20.0%; p < 0.001), and ventilator-associated pneumonia (21.2% *versus* 8.7%; p = 0.01). The administration of muscle relaxants (51.7% *versus* 35.6%; p = 0.02) and aminoglycosides (18.8% *versus* 6.9%, p = 0.01) were also more common among patients with CCI than among the control individuals, and there was a trend towards greater use of corticosteroids among the patients (27.1% *versus* 17.3%; p = 0.09).

**Table 2:** Characteristics of patients after intensive care unit admission

Characteristics	CCI Group(n = 85)	Control Group(n = 115)	p value
SAPS 3	64.6 ±15.1	63.2 ±15.3	0.51
Diagnostic at ICU admission			
Cardiovascular	22 (25.9)	25 (21.7)	0.49
Neurological	22 (25.9)	21 (18.3)	0.19
Respiratory	17 (20.0)	27 (23.5)	0.56
Trauma	10 (11.8)	18 (15.7)	0.43
Surgical	10 (11.8)	15 (13.0)	0.78
Gastrointestinal	3 (3.5)	8 (7.0)	0.29
Other diseases	1 (1.2)	1 (0.9)	0.83
Comorbidities developed before 21 days in the ICU			
Muscle weakness	51 (60.0)	17 (14.8)	<0.001
Pressure ulcers	58 (68.2)	13 (11.3)	<0.001
Sepsis	38 (44.7)	23 (20.0)	<0.001
Acute renal dysfunction	22 (25.9)	19 (16.5)	0.10
Ventilator-associated pneumonia	18 (21.2)	10 (8.7)	0.01
Delirium	9 (10.6)	8 (7.0)	0.36
Unscheduled surgery during ICU stay	24 (28.3)	41 (35.6)	0.26
ARDS	8 (9.4)	7 (6.1)	0.37
Upper gastrointestinal bleeding	3 (3.5)	6 (5.2)	0.57
Candidemia	3 (3.5)	1 (0.9)	0.18
Medications used before 21 days in the ICU			
Neuromuscular relaxant	44 (51.7)	41 (35.6)	0.02
Corticosteroids	23 (27.1)	20 (17.3)	0.09
Aminoglycosides	16 (18.8)	8 (6.9)	0.01
SOFA (day)			
1	7.1 ±3.8	7.4 ±3.6	0.52
7	6.6 ±3.5	5.7 ±3.9	0.12
14	6.2 ±3.1	5.6 ±3.7	0.42
21	5.4 ±3.3	-	-

CCI - chronically critically illness; SAPS 3 - Simplified Acute Physiology Score 3; ICU - intensive care unit; ARDS - acute respiratory distress syndrome; SOFA - Sequential Organ Failure Assessment. Results expressed as mean ± standard deviation or n (%).

### Multivariate analysis and occurrence of chronic critical illness

Table 3 presents three models of multivariate analysis for the prediction of CCI. The global model was designed based on characteristics presented from the time of admission to the time of discharge from the ICU. The independent variables most associated with CCI were the diagnosis of a neurological disease at hospital admission (OR 2.61; 95%CI 1.13 - 6.06; p = 0.03) and the development of pressure ulcers during ICU stay (OR 9.08; 95%CI 4.15 - 25.32; p < 0.001).

**Table 3:** Multivariate analysis of the factors associated with chronic critical illness

Variables	OR (95%CI)	p value
Global analysis		
Age ≥ 65 years	1.57 (0.60 - 4.10)	0.35
Lung disease	1.77 (0.60 - 5.20)	0.29
Chronic renal failure	4.16 (0.80 - 22.0)	0.08
Neurological diagnosis at hospital admission	2.61 (1.13 - 6.06)	0.02
Hospitalization stay prior to ICU admission > 7 days	1.15 (0.48 -2.78)	0.74
Muscle weakness	2.21 (0.88 - 5.55)	0.09
Pressure ulcers	9.08 (4.15 - 25.32)	< 0.001
Sepsis	1.28 (0.51 - 3.21)	0.58
Ventilator-associated pneumonia	2.19 (0.76 - 6.27)	0.14
Neuromuscular relaxant	2.07 (0.92 - 4.63)	0.06
Corticosteroids	0.98 (0.48 - 2.21)	0.63
Aminoglycosides	2.33 (0.68 - 7.92)	0.17
Characteristics prior to ICU admission		
Age ≥ 65 years	1.43 (0.69 - 2.96)	0.94
Chronic lung disease	1.94 (0.86 - 4.37)	0.10
Chronic renal failure	3.57 (1.04 -12.50)	0.04
Neurologic diagnosis at hospital admission	2.25 (1.21 - 4.18)	0.008
Hospitalization stay prior to ICU admission > 7 days	1.10 (0.99 - 1.07)	0.06
Characteristics developed during the ICU stay		
Muscle weakness	2.86 (1.22 - 6.69)	0.01
Pressure ulcers	9.54 (4.07 - 22.35)	< 0.001
Sepsis	1.11 (0.48 - 2.58)	0.79
Ventilator-associated pneumonia	2.15 (0.79 - 5.85)	0.13
Neuromuscular relaxant	1.85 (0.85 - 4.03)	0.11
Corticosteroids	0.82 (0.85 - 4.03)	0.68
Aminoglycosides	1.75 (0.55 - 5.59)	0.34

OR - odds ratio; 95%CI - 95% confidence interval; ICU - intensive care unit.

Based on the findings in table 1, we constructed a second multivariate analysis model that only included the characteristics at hospital admission. Among these characteristics, the variables most associated with CCI were CRF (OR 3.57; 95%CI 1.04 - 12.05; p = 0.04) and a neurological diagnosis as the reason for hospital admission (OR 2.25; 95%CI 1.21 - 4.18; p = 0.008).

The third model of multivariate analysis was constructed based on the findings of table 2 to analyze the association between the conditions developed during ICU stay and the chronification of a critical disease state. The variables that had the strongest associations were the occurrence of muscular weakness (OR 2.86; 95%CI 1.22 - 6.69; p = 0.01) and pressure ulcers (OR 9.54; 95%CI 4.07 - 22.35; p < 0.001).

### Length of stay and mortality

Data on the length of stay and mortality are shown in table 4. Compared with the Control Group, the patients with CCI had a longer stay in the period prior to ICU admission (8.1 ± 10.1 days *versus* 5.1 ± 7.0 days; p = 0.02), in the ICU (29.3 ± 20.4 days *versus* 10.5 ± 6.9 days; p < 0.001) and in the hospital (47.7 ± 29.3 days *versus* 24.6 ± 37.4 days; p < 0.001). No differences were observed in ICU mortality (OR 0.85, 95%CI 0.47 - 1.54, p = 0.29) and hospital mortality (OR 1.33, 95%CI 0.75 - 2.34, p = 0.61). However, among the ICU survivors, the mortality was higher in the CCI Group than in the Control Group (OR 4.30, 95%CI 1.31 - 14.01, p = 0.01).

**Table 4:** Comparison of the groups in relation to the length of stay and mortality

Outcome	CCI Group (n = 85)	Control Group (n = 115)	p value	OR (95%CI)
Length of stay prior to ICU admission (days)	8.1 ± 10.1	5.1 ± 7.0	0.02	-
Length of ICU stay (days)	29.3 ± 20.4	10.5 ± 6.9	< 0.001	-
Length of hospital stay after ICU discharge (days)	12.3 ± 18.3	8.8 ± 14.2	0.33	-
Length of hospital stay (days)	47.7 ± 29.3	24.6 ± 37.4	< 0.001	-
ICU mortality	28 (32.9)	42 (36.5)	0.29	0.85 (0.47 - 1.54)
Hospital mortality in ICU survivors	12/60 (20.0)	4/73 (5.5)	0,01	4.30 (1.31 - 14.01)
Global hospital mortality	40 (47.1)	46 (40.0)	0.31	1.33 (0.75 - 2.34)

CCI - chronically critically illness; OR - odds ratio; 95%CI - 95% confidence interval; ICU - intensive care unit. Results expressed as mean ± standard deviation or n (%).

## DISCUSSION

Our findings reaffirm the high incidence of CCI that has been observed in other studies. For the patients with CCI, the most common reason for ICU admission was neurological diseases, and they had a higher frequency of health care complications, such as pressure ulcers and muscle weakness, than the Control Group.

Patients with CCI present with complex and multifaceted syndromes that cannot be characterized by chronological and arbitrary criteria, such as the duration of MV and/or tracheostomy, and the syndromes should be additionally characterized by clinical and demographic aspects.^(25,27-30)^ We have to consider that longer ICU stays are more likely to result in more acquired issues, and we can assume that this is an inherent characteristic of the population of chronic critical patients classified by chronological criteria. The lack of a definition regarding these criteria led us to adopt the consensus definition for CCI, which is ≥ 21 consecutive days of MV; this definition is still one of the most used criteria and is suggested as the basis for future definitions.^(28)^ It is known that tracheostomy as a criterion of CCI can result in misreports for this population due to the selection of patients in situations different from those associated with chronic diseases.^(7,8)^

Among the subjects admitted to our ICU, 14.8% were classified as having CCI. Despite the adoption of more selective criteria, the occurrence was close to the upper limit of the range reported by other authors (5% to 15%),^(1,6,7,9,10,27)^ which may be explained by the greater severity of disease (mean SAPS > 60) observed in our population.^(7,24)^

For reasons inherent to the physiology of elderly individuals, the recovery of organic insults occurs more slowly; thus, old age is recognized as a risk factor for ICU chronification.^(24,25,27,28)^ However, in our multivariate analysis, age was not identified as a predictor of CCI; the effect of age may have been attenuated by the high proportion of young individuals (less than 1/3 were over 65 years old), which is an expected characteristic in populations treated in trauma hospitals.

Among the characteristics that existed prior to admission to the ICU, CRF as a comorbidity was one of the predictors of CCI. Chronic renal failure is a comorbidity that is associated with other diseases, such as diabetes mellitus and systemic arterial hypertension, with high degenerative potential that increases disease severity and morbidity.^(19,28)^

Neurological diseases at hospital admission constituted the diagnostic category most associated with CCI, corroborating the evidence showing that neurological diseases or abnormalities in the Glasgow coma scale score are the most frequently observed characteristics among patients with CCI.^(3,16,17)^ Carson et al.^(5)^ proposed a multifaceted definition of CCI and suggested that neurological conditions such as ischemic stroke, intracerebral hemorrhages and cranial trauma should be included as criteria for CCI, in addition to prolonged MV, the need for tracheostomy and sepsis.^(3,5)^ It is widely known that sepsis is a prevalent entity in the ICU, and it is associated with high morbidity rates.^(29,31,32)^ Although it was not identified as an important factor in the multivariate analysis, the occurrence of sepsis, as a comorbidity that was developed during ICU stay, among patients with CCI was more than double that observed in the Control Group (44.7% *versus* 20%; p < 0.001) (Table 2). The same result was observed in relation to ventilator-associated pneumonia (21.2% *versus* 8.7%; p = 0.01), a major cause of in-hospital sepsis that is associated with prolonged MV, an intrinsic condition of patients with CCI.^(33-35)^

Regarding the length of hospital stay before admission to the ICU, we detected a statistical tendency that presented it as a predictor of CCI in the multivariate analysis (OR 1.10; 95%CI 0.99 - 1.07; p = 0.06) (Table 3). The length of hospitalization stay prior to admission to the ICU is longer for subjects coming from the ward, who usually present a higher morbidity and mortality in the ICU, than for those referred from the emergency department. These worse outcomes are often associated with prolonged and more severe clinical conditions as well as delays in therapeutic interventions prior to admission to the ICU.^(27)^

Among the characteristics associated with the CCI developed during ICU admission, we identified pressure ulcers and muscle weakness as independent factors (Table 3). Patients with CCI are highly vulnerable to the development of pressure ulcers, usually due to the long stay in the ICU and the long duration of MV. The prevalence can vary between 4% and 49% among critically ill patients, reaching up to 62.5% in those with CCI,^(36)^ a value close to that observed in our subjects (68.2%). Assuming that there is no clear cutoff for the CCI definition and that, for example, some authors consider 14 days as the appropriate cutoff, we might consider that some variables such as pressure ulcers are consequences (even if developed within 21 days) rather than predictors of a chronic condition.

The development of muscular weakness was also associated with CCI and occurred in 60% of our patients, which is consistent with the 30% to 60% rate of occurrence that was reported by other authors.^(37)^ The factors most involved in its occurrence are systemic inflammation, malnutrition, uncontrolled glycemia, sepsis, sedatives, corticosteroids, aminoglycosides, neuromuscular relaxants, immobility and prolonged MV.^(38)^ Many of these factors were confirmed in our results, reinforcing the external validity of our findings. These morbidities may become persistent, affecting patients' prognosis and quality of life in performing activities of daily living.^(29,31,32,34)^

At the end of one year, CCI mortality occurs in 40% to 50% of patients, and less than 10% recover their physical and/or mental autonomy.^(39)^ Thus, the findings of the present study contribute to the understanding of CCI, which can lead to the prevention and treatment of functional disabilities and cognitive sequelae resulting from CCI. The characterization of patients with CCI is of fundamental importance for developing proper management plans for a population of individuals who demand large amounts of resources. Combating CCI begins with prevention in the acute phase of the illness and early goal management strategies, including a spectrum of ventilatory, nutritional, and rehabilitation strategies, that are needed when risk factors are detected.^(24)^ In addition, the results presented here can enrich the body of existing knowledge for the development of stratification and prevention tools for critical illness chronification, as well as plans to provide specialized assistance to these patients.^(29,30)^

Intensive care unit mortality was similar between the two groups, which has also been observed by other authors.^(40)^ The patients' severity according to the SAPS 3 was similar in both groups (CCI Group: 64.6 ± 15.1 *versus* Control Group: 63.2 ± 15.3, p = 0.51), which may have contributed to the similarity in mortality, as well as to a selection bias caused by the fatal outcomes of some patients in the Control Group who might have developed CCI. However, contrary to our expectations, hospital mortality was also similar between groups, which was fundamentally conditioned by the deaths that occurred in the ICU; when the deaths among individuals who survived the ICU were evaluated, the odds of death were much higher among the CCI Group than among the Control Group (OR 4.30, 95%CI 1.31 to 14.01, p = 0.01).

Our study has some limitations. First, it is a study that was carried out in a single hospital with unique characteristics (public, of high complexity and facing problems of availability of ICU beds), which does not allow the extrapolation of the results to other hospital institutions. Second, the observation period was only one year. Third, follow-ups of subjects after hospital discharge were not performed, which restricts the extent to which mortality and the quality of life of patients with CCI can be assessed over time in our study. Fourth, although we reported the success of all extubations, the lack of information on the frequency of reintubations is an additional limitation of the study, considering that the muscular weakness that affects the patient with CCI may interfere in the ventilatory weaning process. Fifth, despite blood transfusions independently contribute to an increased risk for hospital deaths, the length of stay, and costs in critically ill patients undergoing prolonged mechanical ventilation,^(41)^ this factor was not taken into account in our study. Finally, information about care goals for patients in palliative care was not collected, and these patients were not excluded from the analysis.

## CONCLUSION

The incidence of chronic critical illness was high and similar to that observed in other studies. Chronification of the critical illness was strongly associated with chronic renal failure being treated by hemodialysis and diagnoses of neurological diseases at hospital admission, and it was related to complications developed during hospitalization, such as pressure ulcers and muscle weakness. Hospital mortality was high and did not differ from that observed in the control population.
